# 2-Oxo-2*H*-chromen-7-yl 2,2-di­methyl­propionate

**DOI:** 10.1107/S2414314625009496

**Published:** 2025-11-18

**Authors:** Hypolite Bazié, Eric Ziki, Sorgho Brahima, Abdoulaye Djandé, Rita Kakou-Yao

**Affiliations:** a Laboratory of Molecular Chemistry and Materials (LC2M), University Joseph KI-ZERBO, 03 BP 7021 Ouagadougou 03, Burkina Faso; b Laboratory of Matter Environmental and Solar Energy Sciences Research Team: Crystallography and Molecular Physics University Félix Houphouët-Boigny 08 BP 582 Abidjan 08 Ivory Coast; University of Aberdeen, United Kingdom

**Keywords:** crystal structure, coumarin, Hirshfeld surface analysis

## Abstract

In the title compound, C_14_H_14_O_4_, the dihedral angle between the 2*H*-chromen-2-one moiety and the C—CO_2_ ester grouping is 54.30 (5)°. In the crystal, the mol­ecules are linked by C—H⋯O hydrogen bonds forming *C*(6) [100] chains. The contributions to the Hirshfeld surface for the H⋯H, H⋯O/ O⋯H, H⋯C/C⋯H and C⋯C contacts are 49.5, 29.91, 8.6 and 7.7%, respectively.

## Abstract 2

In the title compound, the dihedral angle between the 2*H*-chromen-2-one moiety and the C—CO_2_ ester grouping is 54.30 (5)°. In the crystal, the mol­ecules are linked by C—H⋯O hydrogen bonds forming *C*(6) [100] chains.

## Graphical abstract

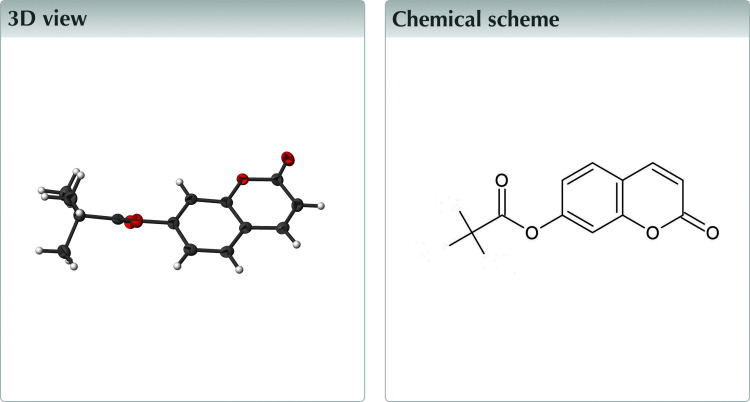

## Structure description

Herein we describe the synthesis, crystal structure and Hirshfeld surface analysis of the title coumarin derivative, C_14_H_14_O_4_. As reported by several authors, coumarin-derived compounds exhibit various biological activities, such as anti­cancer (Yadav *et al.*, 2024 ▸; Rawat *et al.*, 2022 ▸), anti-inflammatory (Todeschini *et al.*, 1998 ▸) and anti-glaucoma (Ziki *et al.*, 2023 ▸) properties.

**Figure 1:**
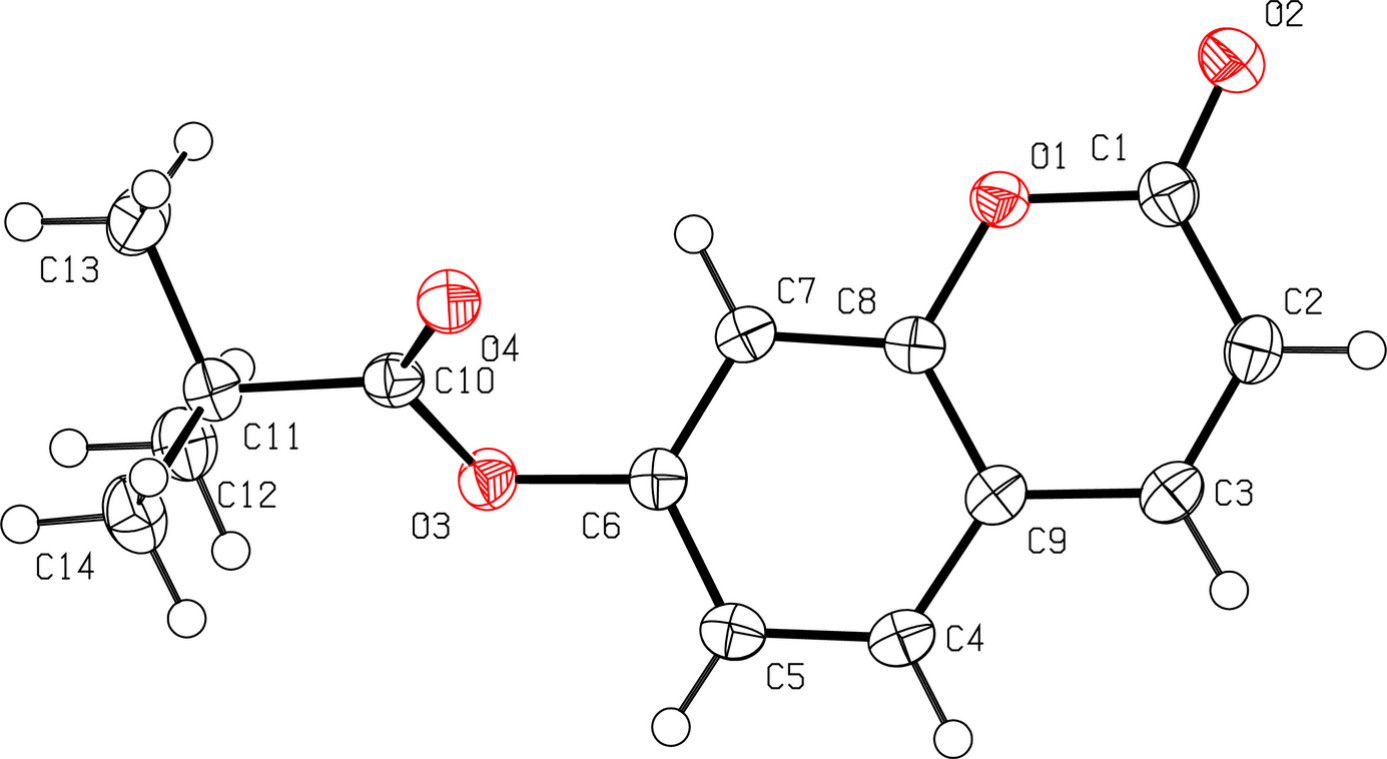
The mol­ecular structure of the title compound with displacement ellipsoids drawn at the 50% probability level.

As expected, the fused ring system formed by atoms C1–C9/O1/O2 is almost planar with an r.m.s deviation of 0.009 Å and the dihedral angle between this ring system and the plane formed by atoms C11/C11/O3/O4 in the ester grouping is 54.30 (5)° (Fig. 1 ▸).

**Figure 2:**
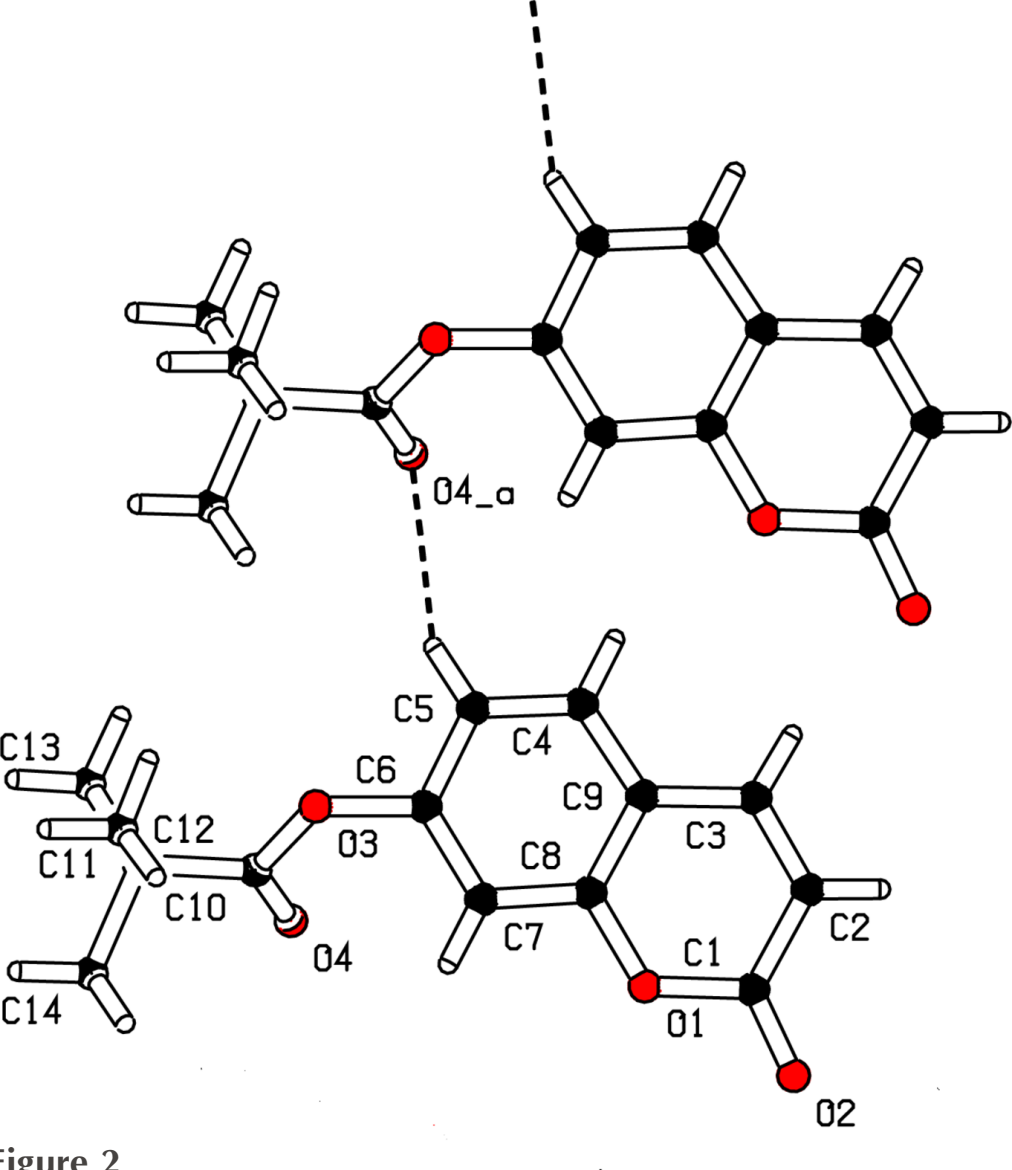
Part of the extended structure of the title compound showing the formation of [100] hydrogen bonded chains. Symmetry code: (*a*) *x* + 1, *y*, *z*.

**Figure 3:**
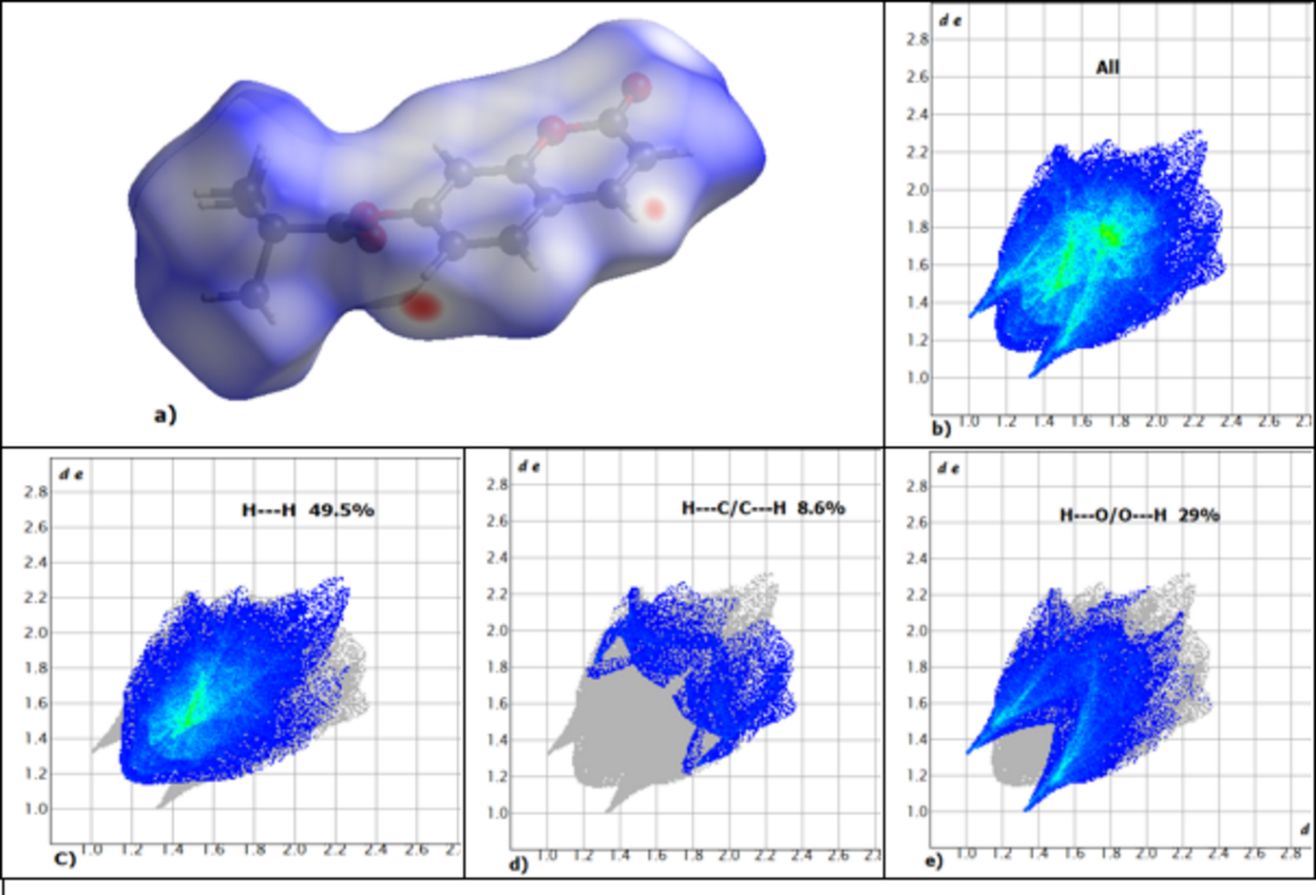
(*a*) Hirshfeld surface of the title compound mapped over *d*_norm_, (*b*) the overall two-dimensional fingerprint plots and (*c*)–(*e*) delineated into contributions from different contacts: H⋯H, H⋯C/C⋯H and H⋯O/O⋯H.

In the crystal, mol­ecules are linked by weak C5—H5⋯O4(*x +* 1, *y*, *z*) hydrogen bonds (Table 1 ▸), thereby generating [100] C(6) chains (Fig. 2 ▸). The Hirshfeld surface and two-dimensional fingerprint plot of the title compound generated by *CrystalExplorer21.5* (Spackman *et al.*, 2021 ▸) confirmed the above inter­action: the C5—H5⋯O4 bond is indicated by the red spots on Fig. 3 ▸*a*. The fingerprint plots show that the most important contributions to the surface are H⋯H and H⋯O/O⋯H contacts with 49.5 and 29.1%, respectively (Fig. 3 ▸*c* and 3*e*). The H⋯C/C⋯H and C⋯C contacts contribute 8.6 and 7.7%, respectively. These values are close to those of 2-oxo-2*H*-chromen-7-yl *tert*-butyl­acetate (Bazié *et al.*, 2025 ▸).

## Synthesis and crystallization

In a 100 ml round-bottom flask equipped with a condenser, pivaloyl chloride (0.76 ml, 6.17 mmol, 1 equiv.) was dissolved in 16 ml of dried diethyl ether and then dried pyridine (2.31 ml, 4.7 equiv.) and 7-hy­droxy­coumarin (1 g, 6.17 mmol, 1 equiv.) were added by small portions over 30 min, with vigorous stirring. The reaction mixture was left stirring at room temperature for 3 h.

The resulting mixture was next poured in a separating funnel containing 40 ml of chloro­form and washed with 5% hydro­chloric acid until the pH was 2–3. The organic phase was extracted, washed with water to neutrality, dried with magnesium sulfate and the solvent removed *in vacuo* until a cloudy solution was obtained. The occurred precipitate while cooling in an ice bath was filtered off with suction, washed with petroleum ether and recrystallized from a chloro­form/*n*-hexane solvent mixture (1:3) giving the title compound as a white powder (0.96 g, yield 63%). Colourless prisms suitable for single-crystal X-ray diffraction analysis were then formed from an acetone solution, after the solvent was left to evaporate slowly at room temperature, m.p. 403–405 K.

## Refinement

Crystal data, data collection and structure refinement details are summarized in Table 2 ▸.

## Supplementary Material

Crystal structure: contains datablock(s) I. DOI: 10.1107/S2414314625009496/hb4533sup1.cif

Structure factors: contains datablock(s) I. DOI: 10.1107/S2414314625009496/hb4533Isup2.hkl

Supporting information file. DOI: 10.1107/S2414314625009496/hb4533Isup3.cml

CCDC reference: 2498840

Additional supporting information:  crystallographic information; 3D view; checkCIF report

## Figures and Tables

**Table 1:** Hydrogen-bond geometry (Å, °)

*D*—H⋯*A*	*D*—H	H⋯*A*	*D*⋯*A*	*D*—H⋯*A*
C5—H5⋯O4^i^	0.93	2.49	3.343 (4)	153

Symmetry code: (i) 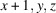.

**Table 2:** Experimental details

Crystal data	
Chemical formula	C_14_H_14_O_4_
*M*_r_	246.25
Crystal system, space group	Triclinic, *P* 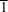
Temperature (K)	296
*a*, *b*, *c* (Å)	6.242 (7), 7.191 (8), 13.652 (16)
α, β, γ (°)	99.05 (6), 92.85 (5), 91.99 (3)
*V* (Å^3^)	603.9 (12)
*Z*	2
Radiation type	Mo *K*α
μ (mm^−1^)	0.10
Crystal size (mm)	0.41 × 0.12 × 0.04

Data collection	
Diffractometer	Bruker D8 Venture
No. of measured, independent and observed [*I* > 2σ(*I*)] reflections	43182, 3718, 2693
*R*_int_	0.056
(sin θ/λ)_max_ (Å^−1^)	0.719

Refinement	
*R*[*F*^2^ > 2σ(*F*^2^)], *wR*(*F*^2^), *S*	0.052, 0.160, 1.09
No. of reflections	3718
No. of parameters	164
H-atom treatment	H-atom parameters constrained
Δρ_max_, Δρ_min_ (e Å^−3^)	0.33, −0.27

Computer programs: *APEX4* and *SAINT* (Bruker, 2019 ▸), *SHELXT2018/2* (Sheldrick, 2015*a* ▸), *SHELXL2018/3* (Sheldrick, 2015*b* ▸), *PLATON* (Spek, 2009 ▸) and *publCIF* (Westrip, 2010 ▸).

## full crystallographic data

### 2-Oxo-2H-chromen-7-yl 2,2-dimethylpropionate Crystal data

**Table:**

C_14_H_14_O_4_	*Z* = 2
*M**_r_* = 246.25	*F*(000) = 260
Triclinic, *P*1	*D*_x_ = 1.354 Mg m^−^^3^
Hall symbol: -P 1	Melting point: 403 K
*a* = 6.242 (7) Å	Mo *K*α radiation, λ = 0.71073 Å
*b* = 7.191 (8) Å	Cell parameters from 3418 reflections
*c* = 13.652 (16) Å	θ = 2.9–30.7°
α = 99.05 (6)°	µ = 0.10 mm^−^^1^
β = 92.85 (5)°	*T* = 296 K
γ = 91.99 (3)°	Prism, colourless
*V* = 603.9 (12) Å^3^	0.41 × 0.12 × 0.04 mm

### 2-Oxo-2H-chromen-7-yl 2,2-dimethylpropionate Data collection

**Table:**

Bruker D8 Venture diffractometer	2693 reflections with *I* > 2σ(*I*)
Radiation source: fine-focus sealed tube	*R*_int_ = 0.056
Mirror monochromator	θ_max_ = 30.7°, θ_min_ = 2.9°
φ and ω scans	*h* = −8→8
43182 measured reflections	*k* = −10→10
3718 independent reflections	*l* = −19→19

### 2-Oxo-2H-chromen-7-yl 2,2-dimethylpropionate Refinement

**Table:**

Refinement on *F*^2^	Secondary atom site location: difference Fourier map
Least-squares matrix: full	Hydrogen site location: inferred from neighbouring sites
*R*[*F*^2^ > 2σ(*F*^2^)] = 0.052	H-atom parameters constrained
*wR*(*F*^2^) = 0.160	*w* = 1/[σ^2^(*F*_o_^2^) + (0.0861*P*)^2^ + 0.0957*P*] where *P* = (*F*_o_^2^ + 2*F*_c_^2^)/3
*S* = 1.09	(Δ/σ)_max_ < 0.001
3718 reflections	Δρ_max_ = 0.33 e Å^−^^3^
164 parameters	Δρ_min_ = −0.27 e Å^−^^3^
0 restraints	Extinction correction: SHELXL-2018/3 (Sheldrick 2015b), Fc^*^=kFc[1+0.001xFc^2^λ^3^/sin(2θ)]^-1/4^
Primary atom site location: structure-invariant direct methods	Extinction coefficient: 0.033 (8)

### 2-Oxo-2H-chromen-7-yl 2,2-dimethylpropionate Special details

**Table:**

Geometry. All esds (except the esd in the dihedral angle between two l.s. planes) are estimated using the full covariance matrix. The cell esds are taken into account individually in the estimation of esds in distances, angles and torsion angles; correlations between esds in cell parameters are only used when they are defined by crystal symmetry. An approximate (isotropic) treatment of cell esds is used for estimating esds involving l.s. planes.

### 2-Oxo-2H-chromen-7-yl 2,2-dimethylpropionate Fractional atomic coordinates and isotropic or equivalent isotropic displacement parameters (Å^2^)

**Table:**

	*x*	*y*	*z*	*U*_iso_*/*U*_eq_	
O1	0.13581 (14)	0.84613 (12)	0.39385 (7)	0.0237 (2)	
O4	0.06432 (15)	0.57567 (13)	0.69115 (7)	0.0269 (2)	
O3	0.36060 (14)	0.76954 (13)	0.72407 (7)	0.0254 (2)	
O2	0.01958 (17)	0.88911 (16)	0.24380 (8)	0.0345 (3)	
C8	0.2943 (2)	0.80064 (17)	0.45874 (9)	0.0217 (3)	
C9	0.4981 (2)	0.75335 (17)	0.42670 (9)	0.0223 (3)	
C7	0.2418 (2)	0.80648 (17)	0.55681 (9)	0.0223 (3)	
H7	0.106957	0.841094	0.577328	0.027*	
C6	0.3981 (2)	0.75876 (17)	0.62275 (9)	0.0227 (3)	
C4	0.6515 (2)	0.70870 (17)	0.49681 (10)	0.0240 (3)	
H4	0.787880	0.677217	0.477166	0.029*	
C10	0.1826 (2)	0.67743 (17)	0.75051 (9)	0.0223 (3)	
C5	0.6034 (2)	0.71067 (18)	0.59473 (10)	0.0242 (3)	
H5	0.705698	0.680589	0.640927	0.029*	
C3	0.5370 (2)	0.75548 (18)	0.32394 (10)	0.0252 (3)	
H3	0.671750	0.727749	0.300907	0.030*	
C1	0.1690 (2)	0.84583 (18)	0.29448 (10)	0.0260 (3)	
C11	0.1609 (2)	0.71001 (18)	0.86250 (10)	0.0257 (3)	
C2	0.3801 (2)	0.79724 (19)	0.26079 (10)	0.0269 (3)	
H2	0.407245	0.794946	0.194259	0.032*	
C13	−0.0793 (2)	0.7139 (2)	0.88096 (11)	0.0338 (3)	
H13A	−0.097988	0.734343	0.951163	0.051*	
H13B	−0.141709	0.814129	0.851933	0.051*	
H13C	−0.148727	0.595837	0.851352	0.051*	
C12	0.2761 (2)	0.8930 (2)	0.91450 (11)	0.0325 (3)	
H12A	0.257507	0.906684	0.984621	0.049*	
H12B	0.426308	0.889022	0.902742	0.049*	
H12C	0.216609	0.998011	0.888773	0.049*	
C14	0.2597 (3)	0.5410 (2)	0.90167 (11)	0.0360 (3)	
H14A	0.249513	0.555599	0.972376	0.054*	
H14B	0.183333	0.426808	0.870998	0.054*	
H14C	0.407801	0.535092	0.886066	0.054*	

### 2-Oxo-2H-chromen-7-yl 2,2-dimethylpropionate Atomic displacement parameters (Å^2^)

**Table:**

	*U*^11^	*U*^22^	*U*^33^	*U*^12^	*U*^13^	*U*^23^
O1	0.0223 (4)	0.0243 (5)	0.0251 (5)	0.0022 (3)	0.0013 (3)	0.0059 (3)
O4	0.0259 (5)	0.0272 (5)	0.0268 (5)	−0.0036 (4)	0.0002 (4)	0.0035 (4)
O3	0.0244 (5)	0.0275 (5)	0.0240 (4)	−0.0028 (4)	0.0014 (3)	0.0045 (4)
O2	0.0309 (5)	0.0431 (6)	0.0320 (5)	0.0033 (4)	−0.0010 (4)	0.0140 (4)
C8	0.0221 (6)	0.0171 (5)	0.0260 (6)	−0.0005 (4)	0.0002 (5)	0.0038 (4)
C9	0.0226 (6)	0.0169 (5)	0.0268 (6)	−0.0018 (4)	0.0032 (5)	0.0015 (4)
C7	0.0212 (6)	0.0195 (5)	0.0264 (6)	0.0006 (4)	0.0027 (5)	0.0035 (4)
C6	0.0249 (6)	0.0192 (5)	0.0236 (6)	−0.0023 (4)	0.0016 (5)	0.0032 (4)
C4	0.0209 (6)	0.0187 (6)	0.0318 (7)	−0.0006 (4)	0.0030 (5)	0.0025 (5)
C10	0.0211 (6)	0.0200 (5)	0.0263 (6)	0.0016 (4)	0.0008 (5)	0.0048 (4)
C5	0.0224 (6)	0.0205 (6)	0.0294 (6)	−0.0008 (4)	−0.0015 (5)	0.0043 (5)
C3	0.0241 (6)	0.0210 (6)	0.0303 (6)	−0.0008 (5)	0.0056 (5)	0.0025 (5)
C1	0.0285 (7)	0.0231 (6)	0.0268 (6)	−0.0008 (5)	0.0015 (5)	0.0062 (5)
C11	0.0278 (6)	0.0261 (6)	0.0232 (6)	0.0011 (5)	0.0009 (5)	0.0042 (5)
C2	0.0302 (7)	0.0250 (6)	0.0256 (6)	−0.0019 (5)	0.0053 (5)	0.0040 (5)
C13	0.0315 (7)	0.0394 (8)	0.0305 (7)	−0.0006 (6)	0.0085 (6)	0.0035 (6)
C12	0.0377 (8)	0.0313 (7)	0.0269 (7)	−0.0007 (6)	−0.0001 (6)	0.0011 (5)
C14	0.0481 (9)	0.0333 (8)	0.0282 (7)	0.0070 (6)	0.0009 (6)	0.0085 (6)

### 2-Oxo-2H-chromen-7-yl 2,2-dimethylpropionate Geometric parameters (Å, º)

**Table:**

O1—C8	1.3772 (19)	C3—C2	1.346 (2)
O1—C1	1.382 (2)	C3—H3	0.9300
O4—C10	1.2058 (19)	C1—C2	1.453 (2)
O3—C10	1.3668 (19)	C11—C12	1.530 (2)
O3—C6	1.405 (2)	C11—C13	1.534 (3)
O2—C1	1.212 (2)	C11—C14	1.539 (2)
C8—C7	1.389 (2)	C2—H2	0.9300
C8—C9	1.402 (2)	C13—H13A	0.9600
C9—C4	1.401 (2)	C13—H13B	0.9600
C9—C3	1.438 (2)	C13—H13C	0.9600
C7—C6	1.383 (2)	C12—H12A	0.9600
C7—H7	0.9300	C12—H12B	0.9600
C6—C5	1.395 (2)	C12—H12C	0.9600
C4—C5	1.383 (2)	C14—H14A	0.9600
C4—H4	0.9300	C14—H14B	0.9600
C10—C11	1.523 (3)	C14—H14C	0.9600
C5—H5	0.9300		
			
C8—O1—C1	121.89 (12)	O1—C1—C2	117.24 (12)
C10—O3—C6	118.83 (11)	C10—C11—C12	112.83 (13)
O1—C8—C7	116.55 (13)	C10—C11—C13	107.60 (12)
O1—C8—C9	121.17 (13)	C12—C11—C13	110.08 (13)
C7—C8—C9	122.28 (12)	C10—C11—C14	106.56 (12)
C4—C9—C8	118.06 (14)	C12—C11—C14	109.61 (14)
C4—C9—C3	124.18 (13)	C13—C11—C14	110.08 (13)
C8—C9—C3	117.76 (12)	C3—C2—C1	121.31 (14)
C6—C7—C8	117.34 (13)	C3—C2—H2	119.3
C6—C7—H7	121.3	C1—C2—H2	119.3
C8—C7—H7	121.3	C11—C13—H13A	109.5
C7—C6—C5	122.65 (14)	C11—C13—H13B	109.5
C7—C6—O3	120.70 (13)	H13A—C13—H13B	109.5
C5—C6—O3	116.50 (12)	C11—C13—H13C	109.5
C5—C4—C9	121.07 (14)	H13A—C13—H13C	109.5
C5—C4—H4	119.5	H13B—C13—H13C	109.5
C9—C4—H4	119.5	C11—C12—H12A	109.5
O4—C10—O3	122.82 (13)	C11—C12—H12B	109.5
O4—C10—C11	124.93 (13)	H12A—C12—H12B	109.5
O3—C10—C11	112.17 (12)	C11—C12—H12C	109.5
C4—C5—C6	118.57 (12)	H12A—C12—H12C	109.5
C4—C5—H5	120.7	H12B—C12—H12C	109.5
C6—C5—H5	120.7	C11—C14—H14A	109.5
C2—C3—C9	120.62 (14)	C11—C14—H14B	109.5
C2—C3—H3	119.7	H14A—C14—H14B	109.5
C9—C3—H3	119.7	C11—C14—H14C	109.5
O2—C1—O1	116.69 (14)	H14A—C14—H14C	109.5
O2—C1—C2	126.05 (15)	H14B—C14—H14C	109.5

### 2-Oxo-2H-chromen-7-yl 2,2-dimethylpropionate Hydrogen-bond geometry (Å, º)

**Table:**

*D*—H···*A*	*D*—H	H···*A*	*D*···*A*	*D*—H···*A*
C5—H5···O4^i^	0.93	2.49	3.343 (4)	153

Symmetry code: (i) *x*+1, *y*, *z*.
